# Army Veterinary Corps

**Published:** 1900-05

**Authors:** 


### ARMY VETERINARY CORPS.

SENATOR KENNEY'S amendment for the establishment of a veterinary corps in the United States army has finally passed the Senate as Section 14 of Senate Bill 4300—a bill for the efficiency of the army, known, however, as the Army Reorganization Bill. This measure is now in the hands of the Military Committee of the House of Representatives for consideration. We have every hope that it will pass and become a law. In our next issue we will be able to give our readers the result, and we will publish the interesting debates which have taken place concerning it.

